# Over-indebtedness and its association with sleep and sleep medication use

**DOI:** 10.1186/s12889-019-7231-1

**Published:** 2019-07-17

**Authors:** Jacqueline Warth, Marie-Therese Puth, Judith Tillmann, Johannes Porz, Ulrike Zier, Klaus Weckbecker, Eva Münster

**Affiliations:** 1 Institute of General Practice and Family Medicine, University of Bonn, Universitätsklinikum Bonn AöR, Sigmund-Freud-Str. 25, 53127 Bonn, Germany; 2 Institute for Medical Biometry, Informatics and Epidemiology (IMBIE), University of Bonn, Universitätsklinikum Bonn AöR, Sigmund-Freud-Str. 25, 53127 Bonn, Germany

**Keywords:** Over-indebtedness, Financial problems, Financial strain, Debt, Sleep problems, Insomnia, Medication use, Hypnotics, Socioeconomic status, Epidemiology

## Abstract

**Background:**

Over-indebtedness is currently rising in high-income countries. Millions of citizens are confronted with the persistent situation when household income and assets are insufficient to cover payment obligations and living expenses. Previous research shows that over-indebtedness increases the risk of various adverse health effects. However, its association with sleep problems has not yet been examined. The objective of this study was to investigate the association between over-indebtedness and sleep problems and sleep medication use.

**Methods:**

A cross-sectional study on over-indebtedness (OID survey) was conducted in 70 debt advisory centres in Germany in 2017 that included 699 over-indebted respondents. The survey data were combined with the nationally representative German Health Interview and Examination Survey for Adults (DEGS1; *n* = 7987). We limited analyses to participants with complete data on all sleep variables (OID: *n* = 538, DEGS1: *n* = 7447). Descriptive analyses and logistic regression analyses were used to examine the association between over-indebtedness and difficulty initiating and maintaining sleep, and sleep medication use.

**Results:**

A higher prevalence of sleep problems and sleep medication use was observed among over-indebted individuals compared to the general population. After adjustment for socio-economic and health factors (age, sex, education, marital status, employment status, subjective health status and mental illness), over-indebtedness significantly increased the risk of difficulties with sleep onset (adjusted odds ratio (aOR) 1.79, 95%-confidence interval (CI) 1.45–2.21), sleep maintenance (aOR 1.45, 95%-CI 1.17–1.80) and sleep medication use (aOR 3.94, 95%-CI 2.96–5.24).

**Conclusions:**

Evidence suggests a strong association between over-indebtedness and poor sleep and sleep medication use independent of conventional socioeconomic measures. Considering over-indebtedness in both research and health care practice will help to advance the understanding of sleep disparities, and facilitate interventions for those at risk.

**Trial registration:**

German Clinical Trials Register: DRKS00013100 (OID survey, ArSemü); Date of registration: 23.10.2017; Date of enrolment of the first participant: 18.07.2017, retrospectively registered.

**Electronic supplementary material:**

The online version of this article (10.1186/s12889-019-7231-1) contains supplementary material, which is available to authorized users.

## Background

Over-indebtedness has been increasing steadily across Europe [1], and beyond [2]. In Germany alone, currently 6.9 million individuals are estimated to be over-indebted, defined as the situation when household income and assets are insufficient to both meet all payment obligations and cover living expenses over a longer period of time [3]. Those affected are facing severe financial stress and are prone to experience social exclusion and stigmatization [4]. While negative consequences of common measures of socioeconomic status (SES), i.e. income, educational attainment and occupation, on health have been well documented [5, 6], over-indebtedness has been largely neglected in health research [7]. Contrary to common belief, however, over-indebtedness is not limited to those in lower socioeconomic positions but may affect individuals at all income and education levels or occupational status [1, 4]. Recent research has revealed that over-indebtedness is a relevant determinant of health independent of conventional socioeconomic measures. Available studies indicate an association between over-indebtedness and mental and physical morbidity [8], such as depression [9], diabetes [10], obesity [11, 12], and back pain [13]. More specifically, a first longitudinal register-based study of 48778 Finnish adults during 1995–2010 has recently shown an association between over-indebtedness and an increased incidence of various chronic diseases [10]. Thus, over-indebtedness has been shown to increase the risk of serious adverse health effects, but its association with sleep problems and associated sleep medication use has not yet been examined.

Recent population studies suggest a steady increase in sleep problems as well as the use of sleep medication in industrialized countries [14–19]. Common sleep disturbances and sleep disorders such as insomnia encompass problems with initiating sleep, remaining asleep, poor sleep quality or insufficient sleep duration [20, 21]. The prevalence rate of these symptoms amounts to approximately 30% whereas specific sleep disorders affect 5 to 10% of adult populations [22–26]. In clinical practice, sedative medications such as hypnotics have a long-standing history in the treatment of sleep problems [27–29]. Between 4 and 10% of adult populations report chronic and current use of sleep medications [29, 30].

Although a growing body of evidence supports an association between physical, psychological as well as socioeconomic parameters and sleep problems and sleep medication use, none of the previous studies has yet considered the potential influence of over-indebtedness.

Sleep is an essential requisite for physical and mental well-being and functioning [21, 56]. Therefore, it is a highly relevant public health issue to address the complexity of socioeconomic disparities in sleep that are assumed to go beyond common measures of socioeconomic status or temporary economic difficulties: Substantial societal cost have been suggested to arise from sleep problems in terms of accidents [57] and occupational injuries [58], absenteeism, productivity [59–66] as well as health care utilization [67, 68]. Moreover, studies have identified associations between sleep problems and a wide range of adverse health effects: Poor sleepers have been shown to have an increased risk of weight gain and obesity [69, 70], hypertension [71–73]; hyperlipidaemia [74, 75]; inflammation [76]; diabetes [71, 74, 77, 78], stroke [75, 79], heart attack [75, 80] mortality [54, 81–83], and reduced quality of life [84].

The aim of this study was to examine the association between over-indebtedness and sleep problems as well as sleep medication use, to contribute to broadening the understanding of underlying mechanisms of sleep disparities. In order to understand the role of over-indebtedness with regards to difficulties with sleep, the prevalence and factors associated with sleep complaints and sleep medication use among over-indebted individuals are assessed, and compared to nationally representative data from the German population.

## Methods

**Fig. 1:**
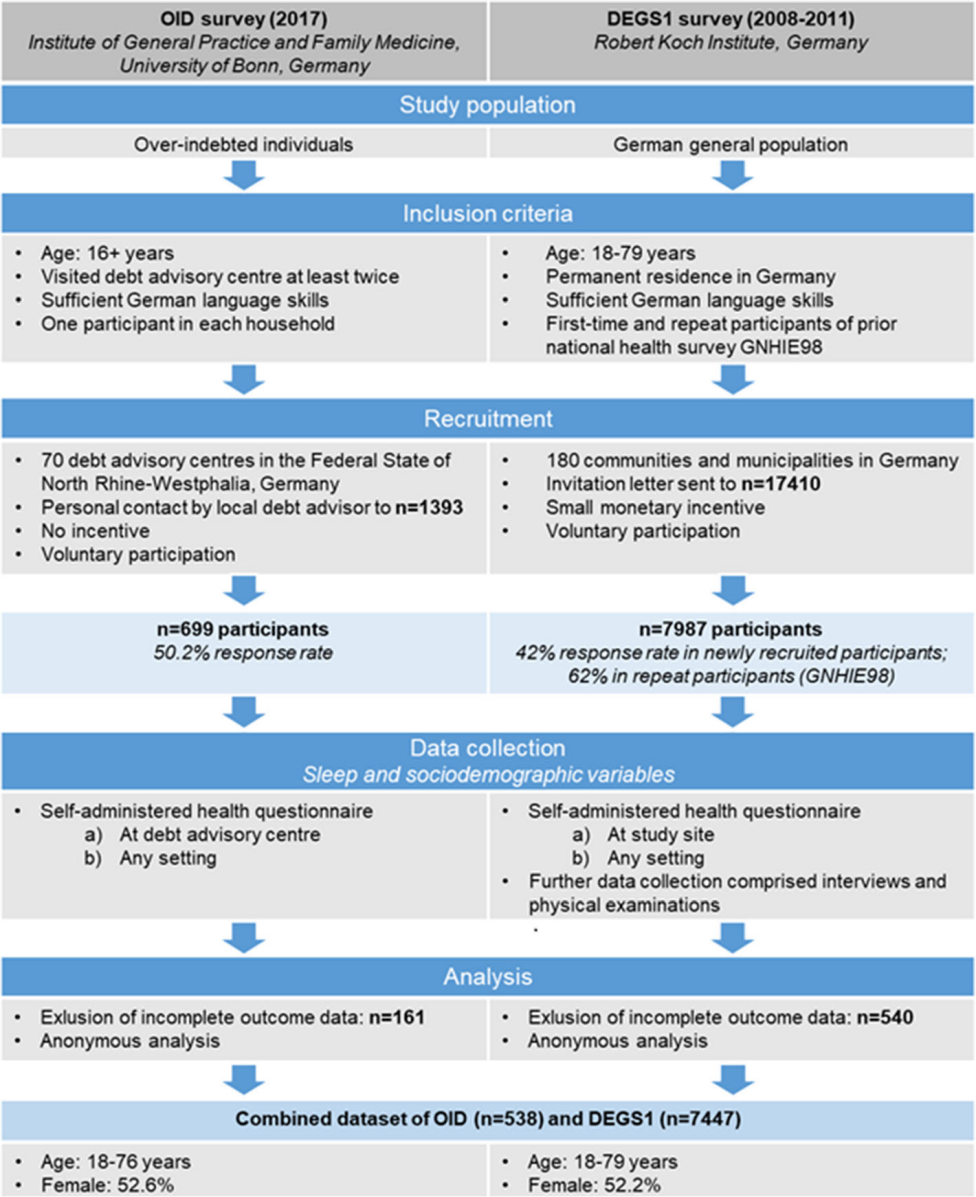
Methods of the OID and DEGS1 survey

The present study is based on a cross-sectional survey among over-indebted individuals (OID survey) [85] that was combined with to the first wave of the German Health Interview and Examination Survey for Adults (DEGS1) [86] (see detailed comparison in Fig. 1).

An anonymous health survey using a self-administered written questionnaire was conducted among clients visiting approved debt advisory centres in North Rhine-Westphalia (NRW), Germany, between July and October 2017. Of 145 non-profit debt advisory centres that were invited to act as recruiters by their umbrella organisation, 70 centres agreed to participate. All debt advisory centres were associated with the local German Consumer Organisation or one of the member organisations of the ‘Expert Committee Debt Counselling of Non-statutory Welfare NRW’ (German: Fachausschuss Schuldnerberatung der Freien Wohlfahrtspflege NRW). Counselling services offered by debt advisory centres across Germany are similar. North Rhine-Westphalia is the most populous of the 16 federal states in Germany (17.5 million inhabitants, 2011). Its demographic structure (gender, age distribution, foreigners) is similar to the national average [87]. We chose to invite advisory centres in North Rhine-Westphalia to participate in the study due to the location of our study centre in that federal state which facilitated contact to both the local umbrella organisations and advisory centres.

Eligibility criteria comprised a minimum age of 16 years due to limited contractual capability. Eligible persons were invited to participate in the survey by debt advisors as of the second consultation because the initial counselling interview reflects a sensitive moment required to build trust. All nationalities were considered but only one respondent within each household. Language, reading and writing skills were required due to the data collection method. Debt advisors received both the study material and a comprehensive information letter which illustrated the study objectives, procedures and eligibility criteria in order to standardise the recruitment and survey. The anonymous self-administered questionnaire that was specifically developed for the target group and stamped addressed envelopes were handed to eligible clients by the debt advisors. The health survey focused on the assessment of medication use and self-medication use among over-indebted individuals. Sociodemographic parameters, including age, sex, education, and measures of over-indebtedness, as well as health status, illnesses and utilization of health care services were assessed. Only respondents who reported sex and age were included for analyses. In the OID survey, a total of 1393 individuals were offered the study material by debt advisors. 699 of these returned the questionnaire with complete data on sex and age which reflects a response rate of 50.2%. This study was approved by the ethical committee of the University Medical Faculty in Bonn (No. 167/17).

The data collected among over-indebted individuals was combined with nationally representative health data. As part of the German health-monitoring programme by the national public health agency, the Robert Koch Institute (RKI), data on adults aged 18 to 79 years was collected in DEGS1 between 2008 and 2011. Methodological considerations of DEGS1 have been published depicting recruitment of participants, data collection and data management in great detail [88, 89]. The random DEGS1 sample selected from local population registries comprised 7987 individuals that were available for public use. While the response rate among newly recruited study participants was 42%, the response rate among adults that had already participated in a previous national health survey in 1997–1999 (German National Health Interview and Examination Survey 1998, GNHIES98) was 62% [88]. The DEGS1 study protocol was approved by the Charité-Universitätsmedizin Berlin ethics committee in September 2008 (No. EA2/047/08).

A broadly accepted definition of over-indebtedness is not yet available. However, over-indebtedness commonly refers to a household’s persistent and ongoing difficulties meeting financial commitments that can be measured by using data on arrears, debt settlement, financial burden or consulting debt counselling services [90]. In our study, we classified all clients of debt advisory centres that were eligible to participate in the survey as over-indebted whereas all participants of the DEGS1 survey were classified as non-over-indebted. Due to the lack of information on debts in the DEGS1 survey, just as in other available population-based surveys, this procedure may introduce attenuation in terms of a bias toward the null. We limited analyses to participants with complete data on all sleep variables (OID: *n* = 538; DEGS1: *n* = 7447). Thus, all participants of the OID survey and DEGS1 with missing data on sleep-related outcome variables, i.e. problems concerning sleep onset, sleep maintenance and/or sleep medication use (OID survey: *n* = 161; DEGS1: *n* = 540) were excluded from analyses. Following the merging of data from the OID survey (*n* = 538) and the DEGS1 survey (*n* = 7447), the combined dataset used for analysis comprised 7985 individuals in total.

Both surveys captured the key outcomes frequency of sleep problems and sleep medication use by the same items: Sleep problems were assessed in terms of difficulties with sleep onset and sleep maintenance in the previous four weeks. The frequency of these sleep problems was rated on a 4-point scale (not at all, less than once a week, once or twice a week, three or more times per week). Likewise, self-reported sleep medication use in the past four weeks was assessed. For logistic regression analysis, the outcome variables were dichotomised referring to the experience of problems with sleep onset, sleep maintenance and sleep medication use (not at all, yes) to assess any complaints related to sleep problems and sleep medication use rather than to identify insomnia disorder.

Based on previous studies, sociodemographic variables were included as covariates in logistic regression analyses to control for potential confounding and systematic differences between the two study populations: Besides sex, age that was classified into four age groups (18–29 years, 30–49, 50–64 and 65–79 years) to differentiate phases of life was considered. Educational level according to the International Standard Classification of Education (ISCED) [91] was classified into three categories (low, medium, high). We dichotomized current employment status as “employed” or “unemployed” to control for occupational factors such as work stress, workload and unemployment that might influence sleep. The data on the current employment status of OID and DEGS1 respondents were derived from multiple answers questions. When participants reported any kind of full or part-time employment, we considered these as currently employed. Marital status was classified into three groups a priori: we compared the married that were cohabiting (Reference) with individuals that were divorced, widowed or living separately and singles to account for potential differences in the frequency of regularly sleeping in a shared bed and the effect of socioeconomic advantages of marriage. Moreover, the subjective health status was included as potential confounder and dichotomised a priori into two groups, i.e. “good” to “very good” versus “fair” to “(very) poor”. The presence of a psychological disorder (absent versus present) was also considered in order to control for the potential impact of such conditions on sleep. In DEGS1, available data on psychological disorders comprised the self-reported 12-month prevalence of diagnosed depression and anxiety disorder. In the OID survey data on both self-reported chronic illnesses and medical indication for medication use in the last seven days was used to capture the presence of a psychological disorder according to the assessment in DEGS1. These data were first classified according to ICD-10-GM (German adaptation of the International Statistical Classification of Diseases and Related Health Problems) by medical professionals. Subsequently, illnesses were further classified to indicate presence or absence of a depression (F32, 33) or anxiety disorder (F40, 41).

Descriptive statistics were used to illustrate population characteristics and to examine differences in the distribution of sociodemographic and health characteristics between the OID and DEGS1 samples using chi-squared test. The prevalence of sleep problems and sleep medication use associated with over-indebtedness was calculated. Multiple logistic regression analysis was used to identify factors that predict sleep problems and use of sleep medication (see Additional file 2: Tables S2 for further analysis). Within covariates missing values that were all below a threshold of 5% were assigned to the most frequent category in the combined dataset of the OID (*n* = 538) and DEGS1 (*n* = 7447) sample. As a sensitivity analysis, we conducted complete case analysis to validate the approach to handle missing data (Additional file 1: Table S1). All independent variables were entered into the model simultaneously. The reference group was defined as the most frequent category, except for the reference category of sex (male) and age (youngest age group) to simplify interpretation. The level of statistical significance was set at 0.05 for all analyses. Analyses were carried out using IBM SPSS statistics (version 25).

## Results

Altogether 7985 persons, aged 18 to 79 years, were included in the combined dataset of the OID (*n* = 538) and DEGS1 (*n* = 7447) samples. Female and male participants were represented in both samples in nearly equal shares (Table 1).

**Table 1:** Study population characteristics and prevalence of sleep problems and sleep medication use, 4 weeks (*n* = 7985)

	OID survey^a^ *n* = 538		DEGS1^b^ *n* = 7447		Differences between samples^†^
	*n*	%	*n*	%	*p*-value
Sex					*p* = 0.846
Female	283	52.6	3885	52.2	
Male	255	47.4	3562	47.8	
Age					*p* < 0.001
18–29 years	103	19.1	1047	14.1	
30–49 years	278	51.7	2444	32.8	
50–64 years	130	24.2	2151	28.9	
65–79 years	27.0	5.0	1805	24.2	
Marital status					*p* < 0.001
Married	112	20.8	4730	63.5	
Separated/Divorced/Widowed	205	38.1	1012	13.6	
Single	214	39.8	1642	22.0	
Missing	7	1.3	63	0.8	
Education level					*p* < 0.001
Low	220	40.9	959	12.9	
Medium	281	52.2	3994	53.6	
High	30	5.8	2473	33.2	
Missing	7	1.3	21	0.3	
Employment status					*p* = 0.927
Not employed	188	34.9	2830	38.0	
Employed	300	55.8	4556	61.2	
Missing	50	9.3	61	0.8	
Depression/anxiety					*p* < 0.001
No	408	75.8	6881	92.4	
Yes	87	16.2	471	6.3	
Missing	43	8.0	95	1.3	
Subjective health status					*p* < 0.001
(Very) good	224	41.6	5539	74.4	
Fair/(very) poor	311	57.7	1878	25.3	
Missing	3	0.6	30	0.4	
Problems with sleep onset					*p* < 0.001
No	149	27.7	3539	47.5	
Yes	389	72.3	3908	52.4	
Problems with sleep maintenance					*p* < 0.001
No	140	26.0	2590	34.8	
Yes	398	74.0	4857	65.2	
Sleep medication use					*p* < 0.001
No	422	78.4	7003	94.0	
Yes	116	21.6	444	6.0	

^a^Over-indebted sample, Germany (2017); ^b^General population sample, Germany (2008–2011)

^†^Chi-squared test

The sample of over-indebted individuals and the DEGS1 sample differed with regards to a number of sociodemographic variables. The over-indebted individuals were significantly younger (70.8% 18–49 years) than the nationally representative sample (46.9% 18–49 years). Educational attainment in the over-indebted was significantly lower whereas the majority of participants in both samples was employed. In contrast to the DEGS1 sample, the majority of over-indebted individuals was not married but single, divorced or widowed (OID survey: 71.4%; DEGS1: 33.9%). Over-indebted individuals had a significantly poorer subjective health status than the general population. Accordingly, the over-indebted population had a significantly higher prevalence of psychological disorders in the form of depression and/or anxiety (16.2%) compared to the general population (6.3%).

### Prevalence of sleep problems and sleep medication use

The prevalence of sleep problems associated with sleep onset (72.3%) and sleep maintenance (74.0%) during the last four weeks was significantly higher among over-indebted individuals than in the general population (52.4 and 65.2%, respectively) (Table 1). Likewise, sleep medication use was significantly more frequent in the over-indebted population (21.6%) than in the general population (6.0%).

**Fig. 2:**
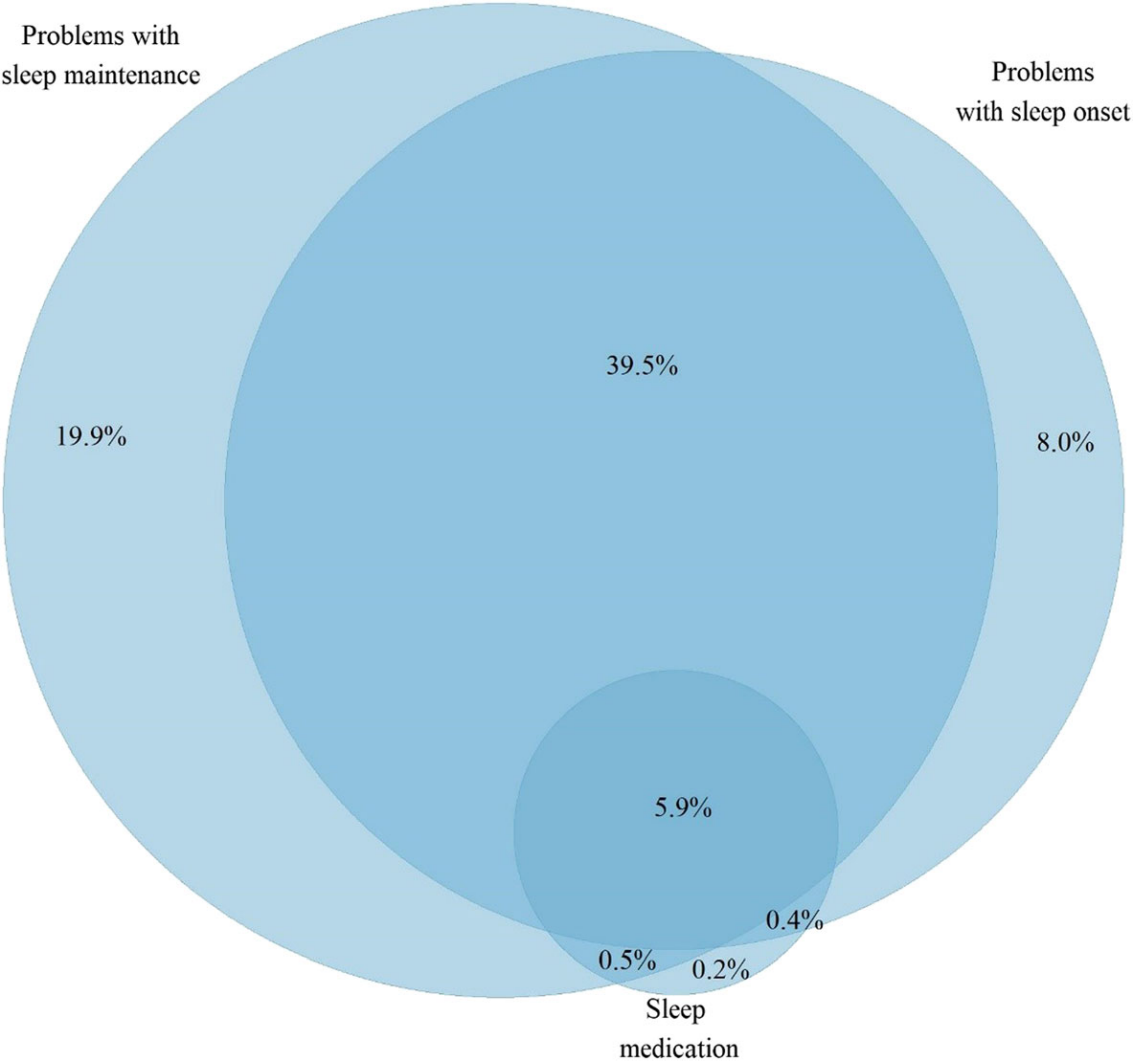
Proportional distribution of sleep problems and sleep medication use (*n* = 7985)

In the combined dataset of OID and DEGS1 participants (*n* = 7985), 39.5% (*n* = 3154) participants reported both problems with sleep onset and sleep maintenance (Fig. 2). Of all participants included in the study, 5.9% (*n* = 471) reported problems with sleep onset and sleep maintenance as well as sleep medication use.

Table 2 illustrates the distribution of sleep problems and sleep medication use by sociodemographic and health characteristics among those affected in the combined dataset of the OID and DEGS1 sample.

**Table 2:** Distribution of sleep problems and sleep medication use by sociodemographic and health characteristics

	Problems with sleep onset		Problems with sleep maintenance		Sleep medication use	
	*n*	%	*n*	%	*n*	%
Total	4297	100.0	5255	100.0	560	100.0
Sex						
Female	2449	57.0	2861	54.4	375	67.0
Male	1848	43.0	2394	45.6	185	33.0
Age						
18–29 years	634	14.8	607	11.6	35	6.3
30–49 years	1350	31.4	1709	32.5	141	25.2
50–64 years	1267	29.5	1648	31.4	176	31.4
65–79 years	1046	24.3	1291	24.6	208	37.1
Marital status						
Married	2476	57.6	3239	61.6	310	55.4
Separated/Divorced/Widowed	741	17.2	881	16.8	149	26.6
Single	1035	24.1	1085	20.6	94	16.8
Missing	45	1.0	50	1.0	7	1.3
Education level						
Low	703	16.4	733	13.9	130	23.2
Medium	2354	54.8	2782	52.9	291	52.0
High	1218	28.3	1716	32.7	135	24.1
Missing	22	0.5	24	0.5	4	0.7
Employment status						
Not employed	2447	56.9	3110	59.2	227	40.5
Employed	1774	41.3	2062	39.2	312	55.7
Missing	76	1.8	83	1.6	21	3.8
Depression/anxiety						
No	484	11.3	540	10.3	174	31.1
Yes	3743	87.1	4631	88.1	372	66.4
Missing	70	1.6	84	1.6	14	2.5
Subjective health status						
(Very) good	2762	64.3	3519	67.0	209	37.3
Fair/(very) poor	1513	35.2	1713	32.6	346	61.8
Missing	22	0.5	23	0.4	5	0.9

Logistic regression analyses evaluated associations between over-indebtedness and sleep problems as well as sleep medication use (Table 3). After adjusting for socio-economic and health factors, including age, sex, education, marital status, employment status, subjective health status and mental illness in the form of depression or anxiety, over-indebtedness significantly increased the risk of sleep problems and sleep medication use.

**Table 3:** Adjusted odds ratios (aOR) and 95% confidence intervals (CI)^†^ of sleep problems and sleep medication use (*n* = 7985)

	Sleep onset		Sleep maintenance		Sleep medication use	
	aOR	95%-CI	aOR	95%-CI	aOR	95%-CI
Over-indebtedness^a^	*1.72*	*1.39–2.13*	*1.39*	*1.12–1.73*	*3.59*	*2.69–4.79*
Sex^b^	*1.48*	*1.35–1.63*	*1.32*	*1.20–1.46*	*1.90*	*1.56–2.31*
Age group						
18–29 years	Reference (Ref.)			Ref.		
30–49 years	0.86	0.73–1.02	*1.27*	*1.07–1.50*	*1.73*	*1.12–2.67*
50–64 years	1.04	0.86–1.25	*1.84*	*1.52–2.22*	*2.61*	*1.66–4.09*
65–79 years	1.04	0.85–1.28	*1.73*	*1.40–2.13*	*4.67*	*2.90–7.51*
Marital status						
Married	Ref.		Ref.		Ref.	
Separated/Divorced/Widowed	1.11	0.97–1.28	1.06	0.92–1.23	0.99	0.78–1.25
Single	*1.26*	*1.10–1.45*	0.98	0.85–1.13	1.05	0.78–1.42
Education level (ISCED)						
Low	0.95	0.82–1.09	*0.79*	*0.68–0.91*	1.21	0.95–1.54
Medium	Ref.		Ref.		Ref.	
High	0.90	0.81–1.00	*1.25*	*1.12–1.39*	1.05	0.84–1.32
Unemployment^c^	1.10	0.97–1.24	0.95	0.84–1.08	1.10	0.88–1.39
Subjective health status^d^	*2.05*	*1.83–2.30*	*1.94*	*1.71–2.20*	*2.61*	*2.14–3.19*
Depression/anxiety^e^	*2.01*	*1.65–2.45*	*2.30*	*1.83–2.88*	*4.39*	*3.50–5.51*

^†^Italics show significant results at alpha = 0.05

^a^Not over-indebted (Ref.) ^b^Male (Ref.); ^c^Employed (Ref.); ^d^Very good to good subjective health status (Ref.); ^e^Absence of depression/anxiety (Ref.)

### Sleep onset

Compared to the general population, over-indebted individuals had a higher risk of problems related to sleep onset whereas the relationship between other socioeconomic factors, i.e. educational attainment or employment status, and difficulty initiating sleep was not significant. Those with a poorer subjective health and/or depression or anxiety had an increased risk of sleep onset problems as compared to those reporting better health or those who did not report these mental disorders, respectively. Female sex was associated with a significantly higher risk of problems with sleep onset. Moreover, singles had a significantly higher risk of difficulty initiating sleep compared to the married.

### Sleep maintenance

Over-indebtedness was also associated with a significantly increased risk of sleep maintenance problems. While individuals with high educational attainment had an increased risk of difficulty maintaining sleep, those with low educational attainment had a lower risk of such sleep problems compared to individuals with a medium education level. The relationship between unemployment and sleep maintenance problems, however, was not significant. Sleep maintenance problems were significantly associated with poorer subjective health and the presence of a psychological disorder. Female sex and age above 29 years were also associated with an increased risk of these sleep problems.

### Sleep medication use

In contrast to over-indebtedness, educational attainment and employment status were not significantly associated with sleep medication use. The use of sleep medication was associated with health factors, i.e. poorer subjective health and the presence of a psychological disorder as well as sociodemographic factors including female sex and age above 29 years.

## Discussion

In the present study, an increased risk of problems with sleep onset and sleep maintenance as well as sleep medication use was observed for over-indebted individuals compared to the general population.

In view of the increasing trend of over-indebtedness of individuals across high-income countries and adverse health effects of inadequate sleep, the study results highlight over-indebtedness as a public health concern. Until today, this is the first study that considers over-indebtedness as a determinant of sleep problems and sleep medication use in health research at the global level. The results suggest that conventional measures of socioeconomic status are insufficient to describe the complexity of financial hardship with regards to its association with health, and sleep specifically.

### Comparison with previous studies

In agreement with previous studies, a high prevalence of sleep problems was found in the present study. Like studies in other high-income countries, sleep problems were more common in women than in men [33], and the share of individuals suffering from disturbed sleep that reported sleep medication use was comparably small [29]. Comorbidity was consistently linked to sleep problems and medication use in this study [31, 32].

#### Financial difficulties

Due to the lack of previous studies on the impact of over-indebtedness on sleep difficulties and sleep medication use specifically, comparability of the present findings is limited. A link between socioeconomic circumstances, typically assessed by education, income and occupational class, and sleep problems has been well established by previous research [46–48, 92, 93]. Evidence on the role of socioeconomic parameters with regards to sleep medication use is rather inconclusive [29, 34, 49, 50]. Lower socioeconomic status has mainly been associated with more frequent sleep problems in terms of general sleep disturbance or specific symptoms (e.g. sleep latency, continuity, duration). Although the prevalence of over-indebtedness is increased in lower socioeconomic positions, however, individuals at all income, education or occupation levels can be over-indebted for various reasons. The small number of available cross-sectional studies and few prospective studies that assess the role of unconventional measures of socioeconomic circumstances illustrate a similar trend: Studies have identified associations between socioeconomic deprivation [53], (ongoing) financial strain [52], as well as past and present economic difficulties [51, 55], and sleep problems. However, socioeconomic indicators including economic difficulties or financial strain are not interchangeable with over-indebtedness.

In contrast to over-indebtedness, less consistent associations of standard socioeconomic measures with sleep problems and sleep medication use were found in the present study. Over-indebtedness, like economic difficulties, affect individuals in all socioeconomic positions but can imply far-reaching psychosocial and legal consequences of unmanageable debt burden for the family, workplace, housing and financial situation that are often long lasting. Although the concept of over-indebtedness exceeds the previously considered spectrum of socioeconomic and psychosocial parameters, available studies indicate that even less severe or short-term financial strain can adversely affect sleep in different ways [94]: In a cross-sectional sample of non-institutionalised elders, self-reported ongoing financial strain that was “somewhat to very upsetting” remained a significant correlate of sleep latency, wakefulness after sleep onset and sleep efficiency measured by polysomnography even after adjusting for sociodemographic and health-related determinants of sleep [52]. In a Finnish working-age sample, past and current economic difficulties were associated with complaints of insomnia, independent of other socioeconomic indicators, whereas education, occupational class and income showed less consistent associations with sleep-related outcomes after adjustment [55, 95]. Accordingly, a cohort study among British and Finnish public sector employees indicated that persistent and increasing economic difficulties, in terms of insufficient financial resources to purchase food and clothes and pay bills, were associated with subsequent sleep problems [51]. In spite of overlapping definitions of economic difficulties and measures of sleep problems, in contrast, a prospective US cohort study of older adults did not identify financial strain as significant predictor of trouble falling asleep or staying asleep but primarily physical health problems and depressed mood [96]. However, the latter study specifically focused on the elderly population and did not assess persistence of financial strain.

With regards to sleep medication use, the role of socioeconomic parameters has not been examined elaborately. A retrospective cross-sectional study of the US population has reported increased odds of sleep medication use not only among individuals with higher educational levels but also the unemployed [34]. In a longitudinal study of a representative US-sample above the age of 50, higher educational attainment was associated with recent sleep treatment utilization but not the use of prescribed sleep medication [50]. Higher income was associated with treatment use outside of a doctor’s recommendation among those currently utilizing treatment. These findings of an association between higher educational attainment and treatment patterns were ascribed to a greater initiative to self-treat among individuals with higher educational level or income [50] and better access to medical information [34]. Use of sleep medication among the unemployed was assumed to relate to sleep disturbances rooted in experiences of anxiety, stress and financial strain [34]. In contrast to the latter findings, a Swiss population-based study found an association between socioeconomic status and subjective and objective measures of sleep but not the use of sleep medication [49]. Crude analyses based on a representative sample of 5000 Norwegian citizens indicated a lower lifetime, current and chronic sleep medication use among those with a higher socioeconomic status in terms of educational level. However, adjusted analyses identified higher socioeconomic status as independent risk factor for higher current sleep medication use [29]. Methodological differences as well as country-specific prescription and payment regulations possibly contribute to the varying results.

#### Psychosocial stress

In line with previous research we assume that mechanisms that link over-indebtedness and sleep outcomes are not only related to material but also psychosocial effects emerging from a persistent lack of financial resources to cover payment obligations and living costs. A reduction of absolute material standards, for instance, in terms of living conditions, can result from accumulating debt over time and facilitate unparalleled experiences of stigmatization, feelings of shame, failure and hopelessness that may induce high levels of stress [41, 42]. Stress exposure, e.g. in terms of upsetting life events, has been suggested as a key trigger of subjective sleep complaints [35, 36]. In line with recent evidence on the influence of hyperarousal (i.e. heightened physiologic or cognitive-emotional activation) on insomnia [43–45], over-indebted individuals’ stress exposure and stress response, in turn, may play a vital role in sleep problems. Over-indebted individuals might feel constrained to increase working hours to cover payment obligations which, in turn, reinforces stress [40, 97], and lead to family conflicts due to challenges juggling family and work. Such work-family conflicts have been shown to be associated with women and men’s sleep complaints [98–100] and sleep medication use among women [101]. Financial strain might also result in relocation to more affordable housing which is typically linked to environmental noise exposure, and might in turn impact sleep [86]. Moreover, stress may affect lifestyle factors such as smoking [102, 103] that has been linked to sleep complaints [37, 38]. In this context, variations in legal regulations and social norms concerning the perception of unmanageable debt across countries [39, 104–107] might contribute to variations in the populations’ stress responses to over-indebtedness.

Although stress is commonly considered a risk factor of disturbed sleep, the evidence for the role of stress in sleep problems is ambiguous. To some extent, this may be due to the wide spectrum of methodological approaches, population characteristics and operationalization of stress and sleep [35, 108]. A number of studies illustrated an association between psychosocial stress and both subjective and objective sleep outcomes in heterogeneous populations [35, 36, 108–115]. In a prospective study among women in the United States, both subjective and objective sleep outcomes were predicted by chronic stress assessed across various domains including work, family as well as finances [35]. On the basis of longitudinal data, Pillai et al. [36] reported a significant relationship between higher levels of stress at baseline in terms of major stressful life events (e.G. *major* illness, divorce or death of a spouse and financial problems) and the onset of insomnia. Greater chronicity of stress exposure was associated with a higher likelihood of developing insomnia. In this context, subjective appraisal of the financial strain and diminished coping abilities that have been linked to sleep disturbances [36, 109] may contribute to an increased susceptibility to insomnia in the face of over-indebtedness. Stress responses in the form of both intentional and involuntary reactions have been shown to significantly mediate the association between stress exposure and insomnia: Maladaptive coping by behavioural disengagement, distraction and substance use as well as cognitive intrusion as a measure of the psychological impact of stressful events were identified as prospective risk factors for insomnia [36]. Moreover, trait sleep reactivity as a measure of sleep disruption in response to stressful events increased the risk for developing insomnia in the same community-based sample of adults with no history of insomnia or depression [116].

Given the severe financial difficulties and psychological stress related to major debt burden, the previous research indicates that over-indebtedness may reflect a relevant risk factor for sleep problems and associated sleep medication use. In line with previous findings, the results of the present study suggest a vital role of over-indebtedness in sleep problems as well as sleep medication use – independent of conventional socioeconomic measures. These findings may, for instance, manifest in barriers to help-seeking for sleep problems from health professionals among those facing severe financial strain. At the same time, potential over- or misuse of sleep medication as a strategy to cope with difficulties initiating or maintaining sleep might contribute to withdrawal or dependence symptoms [117]. Yet further research is necessary to understand the mechanisms between financial difficulties and psychosocial stress related to over-indebtedness, and health outcomes.

### Limitations

Due to the cross-sectional design of the study, the results only reveal associations but does not address causation. However, in line with previous studies [8–13, 118, 119] that have illustrated poor health outcomes in relation to over-indebtedness, it seems more likely that sleep problems reflect an effect of over-indebtedness rather than its cause. Further research is necessary to examine this potential causal link. Recruitment was restricted to those utilizing debt counselling centres more than once which ensures that only those affected by major over-indebtedness that requires further counselling are represented in the OID survey data. Consequently, participants of the OID survey might reflect a subgroup of over-indebted individuals that seek advice due to unbearable strain and associated health effects, including sleep problems, on the one hand. On the other hand, those willing to participate in the survey might also face lower levels of stress as a result of counselling, and in turn report sleep problems less frequently than the target population. Therefore, it cannot be ruled out that the relationship between over-indebtedness and sleep problems and sleep medication use is attenuated or overestimated. Some individuals in the general population sample based on DEGS1 might have been misclassified as non-over-indebted. A possible implication of this is that the association between over-indebtedness and sleep problems and sleep medication use is attenuated.

Differences in the operationalization of variables between the DEGS1 and OID survey may have contributed to biased results. It can be assumed that the assessment of health complaints in the OID survey yields an underrated prevalence of mental illness compared to DEGS1: Over-indebted participants were asked to self-report any chronic illness as well as health complaints underlying medication use in the previous week whereas participants in DEGS1 were specifically asked to self-report certain psychological disorders diagnosed in the previous 12 months. Consequently, effect sizes of over-indebtedness might be overestimated as psychological disorders reflect a vital covariate of sleep outcomes. Concerning data collection, bias might have been introduced by the retrospective assessment of sleep problems and sleep medication use, and errors related to self-reporting.

Exclusion of participants with missing data in one or more sleep outcomes may have introduced bias. However, sensitivity analyses that were conducted to examine the potential influence of exclusion of respondents due to missing data have shown stable results.

Confounding might arise from different sampling and recruitment frames of the two samples compared in this study. However, we adjusted for relevant variables to account for confounding.

Due to a lack of data, a number of possibly relevant covariates that can affect sleep outcomes were not statistically controlled for and may induce unmeasured confounding. For instance, factors such as individual characteristics (e.g. stress response), employment characteristics (shift work, having multiple jobs), caregiving or parenting and ethnicity could not be taken into account, but may confound the association between over-indebtedness and sleep problems and sleep medication use. Ethnicity was assessed equally in both the OID survey and DEGS1, however, not available for public use in DEGS1. Data on income were not collected in the OID survey but educational attainment as well as employment status were considered as standard measures of socioeconomic status previously associated with sleep-related outcomes. Approved debt advisory centres in North Rhine-Westphalia included in this study can be assumed to offer counselling services to clients that have a long tradition and are similar to other regions in Germany. Nevertheless, minor variations in patterns of service use might occur over time and across geographic locations. While these aspects limit the generalizability of our findings, the present study nevertheless provides important evidence on the independent association between over-indebtedness and sleep problems as well as sleep medication use that may be used to guide public health intervention throughout Germany and initiate new lines of research.

## Conclusions

The present study reveals a strong association between over-indebtedness and sleep problems in terms of sleep onset and maintenance as well as sleep medication use. These associations were independent of standard socioeconomic measures and were not accounted for by other sociodemographic or health factors. The results contribute to the increasing evidence on the vital role of socioeconomic factors with regards to sleep problems and sleep medication use. As sleep is essential to good health and functioning, the understanding of risk factors of problems related to sleep is crucial to improve health and reduce health disparities. Considering over-indebtedness as a risk factor for sleep problems helps to provide a basis for early intervention in clinical practice that needs to address both unmanageable debt burden and sleep problems. Specifically, when health-related causes are not detectable, referring over-indebted primary care patients that seek medical consultation for sleep problems to social support and debt counselling services can contribute to alleviating sleep disturbances. Thus, awareness of the association between over-indebtedness and sleep problems and sleep medication use in epidemiologic research and health care can help to avert detrimental health effects and societal cost of sleep problems.

## Additional files

Additional file 1: **Table S1.** Complete case analysis (*n* = 7680) Sensitivity analysis, complete case analysis to validate the approach to handle missing data. (DOCX 16 kb)

Additional file 2: **Table S2.** Adjusted odds ratios (aOR) and 95% confidence intervals (CI) of sleep problems and sleep medication use (*n* = 7985) Additional multiple logistic regression model for sleep problems and sleep medication use, adjusted for sleep medication use and sleep problems respectively. (DOCX 16 kb)

## Data Availability

The data on over-indebted individuals (OID survey) generated and analysed during the current study are not publicly available due to confidentiality concerns but are available from the corresponding author on reasonable request. The general population data (DEGS1) that support the findings of this study are available from the national public health institute, Robert Koch Institute (RKI) (https://www.rki.de/EN/Content/Health_Monitoring/Public_Use_Files/public_use_file_node.html).

## Abbreviations

aOR: Adjusted Odds Ratio
CI: Confidence interval
DEGS1: German Health Interview and Examination Survey for Adults, first wave
GNHIES98: German National Health Interview and Examination Survey 1998
ICD-10-GM: German adaptation of the International Statistical Classification of Diseases and Related Health Problems
ISCED: International Standard Classification of Education
NRW: North Rhine-Westphalia
OID survey: Over-indebtedness-survey
Ref.: Reference
RKI: Robert Koch Institute
SES: Socioeconomic status
