# Supplementary material for: Over-indebtedness and its association with sleep and sleep medication use
Source: BMC Public Health. 2019 Jul 17;19:957. doi: 10.1186/s12889-019-7231-1 (PMC6637586; doi:10.1186/s12889-019-7231-1)
Supplement: Supplementary file 1 — Table S1. Complete case analysis (n = 7680) Sensitivity analysis, complete case analysis to validate the approach to handle missing data. (DOCX 16 kb) [file 12889_2019_7231_MOESM1_ESM.docx]

**Additional File 1**

**Table S1 Complete case analysis (n=7680).**

|  | **Sleep onset** | | **Sleep maintenance** | | **Sleep medication use** | |  |
| --- | --- | --- | --- | --- | --- | --- | --- |
|  | **aOR** | **95%-CI** | **aOR** | **95%-CI** | **aOR** | **95%-CI** |  |
| **Over-indebtedness^a^** | *1.76* | *1.41-2.20* | *1.46* | *1.16-1.84* | *4.05* | *2.98-5.49* |  |
| **Sex^b^** | *1.50* | *1.37-1.65* | *1.33* | *1.20-1.46* | *2.00* | *1.62-2.46* |  |
| **Age group** |  |  |  |  |  |  |  |
| 18-29 years | Reference (Ref.) | | Ref. |  | Ref. |  |  |
| 30-49 years | 0.89 | 0.75-1.06 | *1.31* | *1.10-1.56* | *1.75* | *1.11-2.75* |  |
| 50-64 years | 1.10 | 0.91-1.33 | *1.91* | *1.57-2.32* | *2.77* | *1.73-4.44* |  |
| 65-79 years | 1.06 | 0.86-1.31 | *1.80* | *1.45-2.24* | *4.47* | *2.71-7.37* |  |
| **Marital status** |  |  |  |  |  |  |  |
| Married | Ref. |  | Ref. |  | Ref. |  |  |
| Separated/Divorced/ Widowed | 1.12 | 0.97-1.29 | 1.06 | 0.91-1.23 | 0.97 | 0.76-1.24 |  |
| Single | *1.33* | *1.15-1.53* | 1.01 | 0.88-1.18 | 1.07 | 0.78-1.46 |  |
| **Education level (ISCED)** |  |  |  |  |  |  |  |
| Low | 0.94 | 0.82-1.09 | 0.79 | 0.68-0.91 | 1.21 | 0.94-1.57 |  |
| Medium | Ref. |  | Ref. |  | Ref. |  |  |
| High | 0.90 | 0.81-1.00 | *1.24* | *1.11-1.38* | 1.14 | 0.90-1.44 |  |
| **Unemployment^c^** | 1.12 | 0.99-1.27 | 0.96 | 0.84-1.09 | 1.23 | 0.97-1.57 |  |
| **Subjective health status^d^** | *2.02* | *1.80-2.27* | *1.94* | *1.70-2.20* | *2.64* | *2.14-3.25* |  |
| **Depression/anxiety^e^** | *2.04* | *1.67-2.49* | *2.39* | *1.89-3.03* | *4.35* | *3.43-5.52* |  |

^†^Italics show significant results at alpha = 0.05.

^a^Not over-indebted (Ref.) ^b^Male (Ref.); ^c^Employed (Ref.); ^d^Very good to good subjective health status (Ref.); ^e^Absence of depression/anxiety (Ref.)
