# Supplementary material for: Over-indebtedness and its association with sleep and sleep medication use
Source: BMC Public Health. 2019 Jul 17;19:957. doi: 10.1186/s12889-019-7231-1 (PMC6637586; doi:10.1186/s12889-019-7231-1)
Supplement: Supplementary file 2 — Table S2. Adjusted odds ratios (aOR) and 95% confidence intervals (CI) of sleep problems and sleep medication use (n = 7985) Additional multiple logistic regression model for sleep problems and sleep medication use, adjusted for sleep medication use and sleep problems respectively. (DOCX 16 kb) [file 12889_2019_7231_MOESM2_ESM.docx]

**Additional File 2**

**Table S2 Adjusted odds ratios(aOR) and 95% confidence intervals(CI)^†^ of sleep problems and sleep medication use (n=7985).**

|  | **Sleep onset** | | **Sleep maintenance** | | **Sleep medication use** | |
| --- | --- | --- | --- | --- | --- | --- |
|  | **aOR** | **95%-CI** | **aOR** | **95%-CI** | **aOR** | **95%-CI** |
| **Over-indebtedness^a^** | *1.54* | *1.24-1.91* | *1.26* | *1.01-1.58* | *3.12* | *2.32-4.20* |
| **Sex^b^** | *1.43* | *1.30-1.57* | *1.28* | *1.16-1.41* | *1.68* | *1.37-2.05* |
| **Age group** |  |  |  |  |  |  |
| 18-29 years | Reference (Ref.) | | Ref. |  | Ref. |  |
| 30-49 years | 0.85 | 0.71-1.00 | *1.25* | *1.06-1.48* | *1.75* | *1.13-2.72* |
| 50-64 years | 1.00 | 0.83-1.20 | *1.78* | *1.48-2.15* | *2.42* | *1.53-3.82* |
| 65-79 years | 0.94 | 0.77-1.16 | *1.60* | *1.30-1.98* | *4.60* | *2.83-7.47* |
| **Marital status** |  |  |  |  |  |  |
| Married | Ref. |  | Ref. |  | Ref. |  |
| Separated/Divorced/  Widowed | 1.11 | 0.96-1.27 | 1.06 | 0.91-1.22 | 0.94 | 0.74-1.20 |
| Single | *1.26* | *1.09-1.44* | 0.97 | 0.84-1.12 | 0.99 | 0.73-1.34 |
| **Education level (ISCED)** |  |  |  |  |  |  |
| Low | 0.93 | 0.81-1.07 | *0.77* | *0.67-0.89* | 1.28 | 1.00-1.64 |
| Medium | Ref. |  | Ref. |  | Ref. |  |
| High | 0.90 | 0.81-1.00 | *1.24* | *1.11-1.39* | 1.05 | 0.83-1.32 |
| **Unemployment^c^** | 1.09 | 0.97-1.24 | 0.95 | 0.84-1.08 | 1.05 | 0.83-1.33 |
| **Subjective health status^d^** | *1.92* | *1.71-2.15* | *1.82* | *1.61-2.07* | *2.09* | *1.71-2.57* |
| **Depression/anxiety^e^** | *1.67* | *1.37-2.04* | *1.97* | *1.56-2.48* | *3.80* | *3.01-4.80* |
| **Sleep medication use^f^** | *5.56* | *4.19-7.38* | *4.12* | *3.02-5.62* | – |  |
| **Problems with sleep onset^g^** | – |  | – |  | *4.31* | *3.20-5.79* |
| **Problems with sleep maintenance^h^** | – |  | – |  | *2.37* | *1.71-3.28* |

^†^Italics show significant results at alpha = 0.05.

^a^Not over-indebted (Ref.) ^b^Male (Ref.); ^c^Employed (Ref.); ^d^Very good to good subjective health status (Ref.); ^e^Absence of depression/anxiety (Ref.); ^f^No sleep medication use; ^g^Absence of problems with sleep onset (Ref.); ^h^Absence of problems with sleep maintenance (Ref.)
